# Gleanings in Science and Medicine

**Published:** 1893-09-02

**Authors:** 


### Gleanings in Science and Medicine.

Professor A. McAllister took the opportunity during a recent lecture of reading to his audience a translation of what is supposed to be the oldest subscription now existing among "Medical Papyri." This particular specimen dates back 4000 B.C., and was at that time intended as a hair wash recipe for the mother of King Chata, second King of the First Dynasty, and the prescription reads as follows: "Pad of a dog foot, the hoof of an ass, and the fruit of a date palm. Take of these equal proportions, and boil together in oil in a saucepan. Directions for use: Rub thoroughly in."

The prolonged drought has been responsible for many natural phenomena, but we were not prepared to attribute to it as a result any value of an archæological order, yet such has been the case, in one instance at any rate, at Long Whittenham, in Berkshire. Here the dryness of the earth's surface, through causing deep cracks and fissures, has recently led to the discovery of the hitherto unsuspected existence of the remains of an ancient Roman camp. Strange and bizarre as this result of our extraordinary summer, there are yet other matters which, though more purely within the weather's ordinary province, are scarcely less remarkable. Summer is this year began in March, when that leonine month gave us days as mild as those of an ordinary June. On February 16th a blackbird's, and on the 18th a throstle's nest were found, not only built, but with several eggs respectively in each, whilst in early March a robin's nest with young was discovered; and in the following month the swallow had laid her eggs. By May time strawberries were ripe in many exposed situations, and cherries gathered before June. Spring, indeed, forestalled summer this year, whilst this latter season has exceeded the records of the Jubilee year in length as in heat.

The abnormally low price of English corn, and the plethora of foreign grain which finds its way into our markets, is causing us little by little to turn all available arable lands into pasture grounds. Under the circumstances, the suggestion occurs to us, why should not we English follow the example set us by the Swiss, which nation have turned their grass lands to such good account, that in one small locality alone no fewer than 14,365 cows are yearly grazed. This, indeed, has been effected at the little town of Cham, known as the headquarters of the tinned milk trade, in which commodity the inhabitants are carrying on a world-wide business. They are an industrious people, the Swiss, a race ever ready to turn small advantages into greater; it was only a little waste ground and a few cows which first gave rise to a business which has since assumed proportions of a gigantic scale. But why should not we English take an example from a thrifty race, anyhow as regards this simple industry, and if our ambition does not soar into a greater international sphere, that surely is no reason why we should not supply our own market with the commodity on which the little Swiss town so long has flourished.

